# Disulfide-induced self-assembled targets: A novel strategy for the label free colorimetric detection of DNAs/RNAs via unmodified gold nanoparticles

**DOI:** 10.1038/srep45837

**Published:** 2017-04-07

**Authors:** Ehsan Shokri, Morteza Hosseini, Mehdi D. Davari, Mohammad R. Ganjali, Maikel P. Peppelenbosch, Farhad Rezaee

**Affiliations:** 1 Department of Life Science Engineering, Faculty of New Sciences & Technologies, University of Tehran, Tehran, Iran; 2 Lehrstuhl für Biotechnologie, RWTH Aachen University, 52056 Aachen, Germany; 3 Center of Excellence in Electrochemistry, Faculty of Chemistry, University of Tehran, Tehran, Iran; 4 Biosensor Research Center, Endocrinology & Metabolism Molecular-Cellular Sciences Institute, Tehran University of Medical Sciences, Tehran, Iran; 5 Department of Gastroenterology and Hepatology, Erasmus Medical Center, Rotterdam, The Netherlands; 6 Department of Cell Biology, University of Groningen, University Medical Center Groningen, Groningen, The Netherlands

## Abstract

A modified non-cross-linking gold-nanoparticles (Au-NPs) aggregation strategy has been developed for the label free colorimetric detection of DNAs/RNAs based on self-assembling target species in the presence of thiolated probes. Two complementary thiol- modified probes, each of which specifically binds at one half of the target introduced SH groups at both ends of dsDNA. Continuous disulfide bond formation at 3′ and 5′ terminals of targets leads to the self-assembly of dsDNAs into the sulfur- rich and flexible products with different lengths. These products have a high affinity for the surface of Au-NPs and efficiently protect the surface from salt induced aggregation. To evaluate the assay efficacy, a small part of the citrus tristeza virus (CTV) genome was targeted, leading to a detection limit of about 5 × 10^−9^ mol.L^−1^ over a linear ranged from 20 × 10^−9^ to 10 × 10^−7^ mol.L^−1^. This approach also exhibits good reproducibility and recovery levels in the presence of plant total RNA or human plasma total circulating RNA extracts. Self-assembled targets can be then sensitively distinguished from non-assembled or mismatched targets after gel electrophoresis. The disulfide reaction method and integrating self-assembled DNAs/RNAs targets with bare AuNPs as a sensitive indicator provide us a powerful and simple visual detection tool for a wide range of applications.

Colorimetric assays have attracted a great deal of attention because of the simple, speedy and a direct visual detection methods they offer without the need for any complicated equipment^1^,^2^. Gold nanoparticles (GNPs) have been used as the sensing material for colorimetric detections due to their unique optical properties and ease of surface modifications^3^,^4^. The optical properties of AuNPs correspond to a phenomenon known as localized surface plasmon resonance (LSPR), which originates from the resonance collective oscillation of valence electrons in the conduction band of metallic nanoparticles, upon interacting with an electromagnetic field^5^. AuNPs’ LSPRs usually occur between 520 and 800 nm and the spectral position of the LSPR band is strongly dependent on the particle size and shape. The bright colors of AuNPs, which is the basis for their application as markers, is due to the presence of a plasmon absorption band, which is formed when the frequency of the incident photon resonates with the collective excitation of the conductive electrons of the particle^6^. The surface plasmon absorption peak for GNPs is very sensitive to the environment, shifting towards longer wavelengths (red shift) and broadening significantly upon GNP aggregation^7^. This is a direct result of electric dipole–dipole interactions and couplings between the plasmons of the neighboring particles in the aggregates formed^6^. The interparticle plasmon coupling leads to a huge absorption band shift (up to ≈300 nm). Consequently, color changes can be observed by naked eye and can be used for the qualitative analyses without sophisticated instruments^8^,^9^. The color shift of AuNPs colloids occurs in both directions according to the inter-particle distance changes from dispersed to the aggregate state (blue shift) or the reverse (red shift). The aggregation/dispersion extents are proportional to the absorption peak shift, so the signal is quantifiable and provides a direct measure of the aggregation- or separation-inducing agent (e.g., an active enzyme, DNA, etcetra)^7^. To date, a large number of developed methods used AuNPs-based colorimetric assays for the detection of DNA/RNA sequences of which two of the most common strategies used frequently for colorimetric detection are 1-cross-linking and 2-non-cross-linking aggregation. The former approach was introduced by Mirkin’s team. In this system, AuNPs capped with 3′- and 5′-(alkanethiol) oligonucleotides are used to complex a specific DNA target. The hybridization of the DNA target with two sets of DNA-modified AuNPs results in the formation of AuNPs/polynucleotide network, which triggers a red to purple color change in solution^10^,^11^. In such assays, the target nucleic acid acts as a cross-linker between two DNA-functionalized AuNPs. DNA-templated self-assembly binary of AuNPs has become a new area of research in the field of bio-nanotechnology and, up to now, it has been used to prepare new types of biomaterials, nanodevice and biosensors^12^. Recently, the reverse process based on redispersion of the DNA-linked AuNP aggregates has also been shown to be one effective approach for developing colorimetric bioassays. To control the disassembly of DNA-linked AuNPs, the different stimuli have been well studied such as temperature^13^, enzymes^14^, metal ions^15^ and small molecules^16^. However, as reported by Trantakis and co-workers, DNA detection based on target-induced disassembly in the absence of toeholds remained quite slow; a long time consuming of more than 6 hours was required to be completed^17^. More recently, Lam *et al*. conducted a DNA detection experiment based on DNA-linked AuNPs via a strand displacement mechanism^18^. These authors were able to decrease the rate of AuNPs disassembly by a fine-tuning the toehold length and assay temperature^18^.

The non-cross-linking aggregation is based on using unmodified or bare AuNPs. Li and Rothberg found that single-stranded (ss) DNA can bind to citrate-capped AuNPs through DNA base–gold interactions and electrostatically stabilize AuNPs. In the presence of the complementary DNA targets, the formed duplex structure is not as robust as ssDNA and shows a low binding affinity to citrate-capped AuNPs. So, at an appropriate salt concentration AuNPs are stabilized in the presence of ssDNA, but aggregate in the presence of dsDNA^19^. They applied this rule to the specific diagnosis of both DNA^12^,^20^ and mRNA^21^. However, due to the low sensitivity of this method, a step of DNA amplification by PCR before detection was inevitable^22^. Latter, other groups illustrated the discrimination of wild type from mutant cell lines by applying the same method^23^. Other investigators also improved the sensitivity of the detection assay based on the concept that both ssDNA and dsDNA have the ability to stabilize AuNPs at low salt concentrations^20^,^24^. In this respect, Xia *et al*.^24^ used a positive charge polyelectrolyte to sequester ssDNA probes in the solution assay. Thus in the absence of target, ssDNA probes cannot protect AuNPs from aggregation against low salt concentrations. On the other side, the polyelectrolyte has no effect on dsDNA formed in the presence of the target. In such state, the AuNPs remain stable and monodispersed upon its addition^24^.

Despite the advantages of both cross-linking and non-cross-linking aggregation applications for colorimetric biosensing and DNA analysis, several challenges have been addressed on the limits to the widespread use of these technologies in previous reports. First, interparticle cross-linking aggregation suffer several drawbacks such as: (i) the oligonucleotides need to be chemically immobilized on the AuNPs, (ii) the original concentration of the nanoparticles is lost during centrifugation and work-up (Approximately 25–30%)^9^, (iii) a long time is required between the particles modification stage and the start of the analysis (about 4.5 days)^18^, (iv) tedious and often unsuccessful salt-aging processes have to be performed, (v) different functionalized AuNPs are required for different targets (the heterogeneity effect), (vi) to discriminate full and mismatch sequences melting analyses need to be run^9^,^25^ (vii) loading DNAs on the surface of particles strongly depends on the oligonucleotide base composition (spacer, linker and overhange should be used)^26^, (viii) it is necessary to optimize the variables influencing the uniform and reliable loading of DNA (i.e. the effects of salt concentration, spacer composition, nanoparticle size, surfactant, etc.). Second, the main problems of the non-cross-linking aggregation or unmodified AuNPs based DNA biosensors includeing sensitivity to the local environment, non-specific response, instability and in particular complex real samples^27^. In such system, it is also shown that adsorption of DNA on AuNPs surface is a function of length and temperature^22^.

To deal with these issues of concerns, the present study was designed with focuses on the development of a novel and simple strategy based on the use of thiolated primers and unmodified AuNPs for colorimetric detection of DNAs/RNAs targets. According to this strategy, frst the 3′- and 5′- thiol modified oligonucleotide probes simultaneously recognize their specific DNA targets on both tails. Next continuous disulfide bond formation at 3′ and 5′ terminals leads to the growth and self-assembly of dsDNA on both sides into the sulfur-rich supramolecular structures with different lengths. Mixing these products with an unmodified AuNPs solution was found to be resulted in shielded particles, which were more salt-tolerating than AuNPs linked to disulfide-interconnected probes (Fig. 1). In this regard, a facile colorimetric method for the detection of DNAs/RNAs in aqueous or biological samples by naked eye was achieved without time-consuming chemical modifications, expensive instruments and experienced operators. Additionally, the method was found to be sensitive to DNA nucleotide polymorphisms and the detection range could be tuned up by different concentrations of the probes. The viability of this simple colorimetric detection system was put to test through using it in the diagnosis of the *Tristeza* virus and miRNA 118 in plant total RNAs and human plasma circulating total RNA respectively.

## Results and Discussion

### Principle

**Figure 1:**
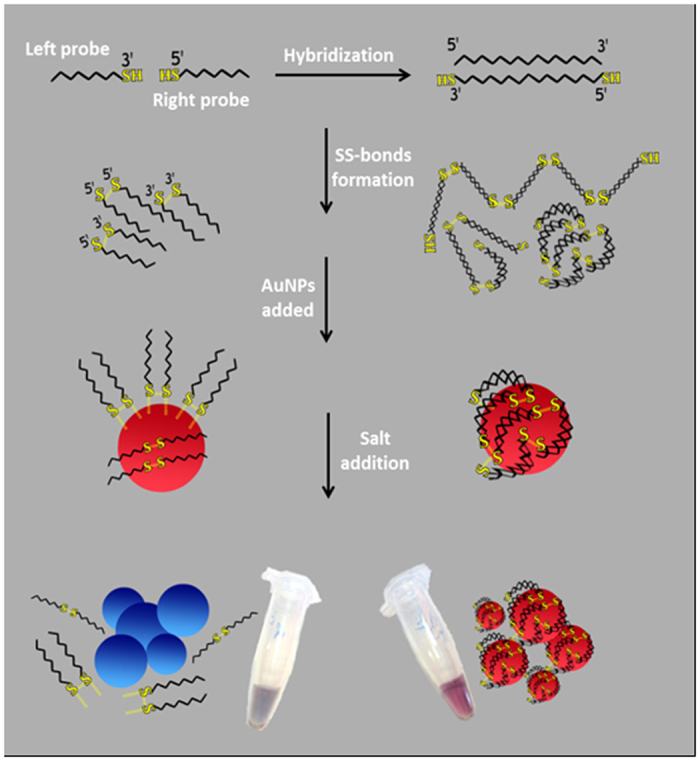
Schematic illustration of the overall colorimetric strategy based on unmodified AuNPs using disulfide induced self-assembling of DNA targets in the presence of left and right thiolated probes.

The principle of the proposed colorimetric approach based on the application of un-modified AuNPs for sensitive detection of DNA/RNA targets via the disulfide induced self-assembly mechanism is shown in Fig. 1. To examine the process in details, a small region (30 mers) of citrus tristeza virus (CTV) severe strain’s genome was selected as a target (Table 1). The CTV, a member of the genus Closterovirus in the family Closteroviridae, has been responsible for the death or total loss of production of about 100 million citrus trees over the past century. It causes three major diseases, depending on the virus strain, host species, and scion/rootstock combination^28^. The virus exists as multiple types that usually are referred to as mild and severe strains^29^. Trees affected by severe strains become devalued economically due to low yield and poor fruit quality.

The experiment begins with incubating the targets (DNAs/RNAs) and thiol modified probes (3′ L and 5′ R) in 20 mM of a phosphate buffer (pH 7.0) at 90 °C for 10 min. Subsequently, the mixture was gradually cooled to room temperature to reach the annealing temperature at which the 3′ L and 5′ R probes recognize the specific targets on the both ends and then stored additionally for at least 4 h at room temperature in order to generate the formation of disulfide bonds. The continuous disulfide bond formation at 3′ and 5′ terminals leads to the self-assembly of dsDNA on both sides and sulfur rich supramolecular structures with different lengths. The resulting mixtures were then treated with unmodified AuNPs for 30 minutes and subsequently exposed to high amounts of a salt (300 mM MgCl_2_). As a result, the sulfur rich self-assembled dsDNAs was more flexible and longer than single dsDNA that effectively bound to the AuNPs, no aggregation occurred, and the solution maintained its original red color. However, in the absence of targets, the self- assembled structures did not form because there was no template for the probes to attach to it. In this state, left and right thiolated probes were connected to themselves or other probes via disulfide bridges and formed a batch of short homodimers and hetrodimers. These structures, due to the rigidity and short length, cannot effectively wrap around the AuNPs as self-assembled dsDNAs, leading to their aggregation in the high-ionic strength (Fig. 1).

**Figure 2:**
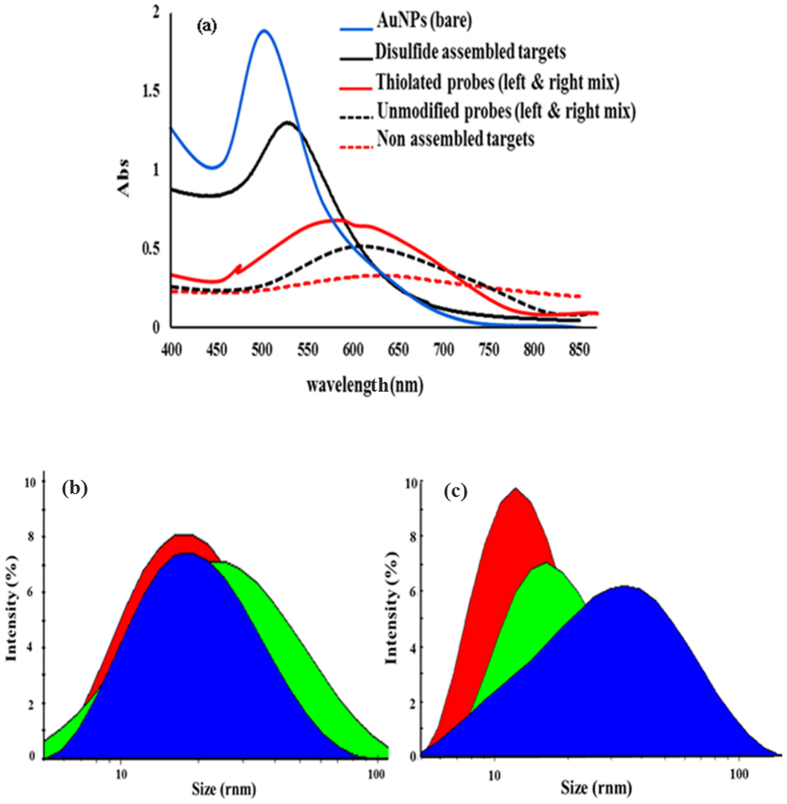
(**a**) UV-Vis spectra of the AuNPs suspension treated with salt in the presence of assembled and non assembled DNA targets. The effects of unmodified and thiolated probes on the absorption intensity were also shown. (**b**) DLS graph of AuNPs size distribution in the presence of unmodified probes and non assembled targets. (**c**) DLS graph of AuNPs size distribution in the presence of thiolated probes and self-assembled targets. Red: citrate-capped AuNPs, Green: AuNPs mix with modified or unmodified probes, Blue: AuNPs mix with assembled or non assembled DNA targets.

In order to obtain a more detailed knowledge of the method, UV-visible spectrophotometry was used. The changes in the absorption of the AuNPs solutions merely containing thiolated probes (the blank sample) or in the presence of target (CTV severe DNA, Test sample), were recorded 10 minutes after addition of MgCl_2_. In addition, the CTV severe DNA was allowed to hybridize with unmodified probes (3′ UL & 5′ UR) to make it possible to compare the stabilizing power of disulfide assembled with non-assembled targets in the AuNPs solution. As shown in Fig. 2a, the absorption band of bare AuNPs at 520 nm was decreased and slightly shifted to 530 nm (in the presence of the assembled targets). In this regard, the color of the solution remained red and AuNPs colloids were still stable and continued dispersed in the solutions. However, the samples treated with non-assembled targets showed a very broad band with a concomitant red-to-colorless change (Fig. 2a). This suggests that the non-assembled targets due to short lengths (30 mers) and double helix rigidity cannot cap and stabilize the AuNPs under high salt condition. In contrast, the dispersion of the AuNPs, in the presence of disulfide assembled dsDNA targets, was attributed to the formation of long assemblies of dsDNA, which were more flexible (as a function of dihedral angles and internal rotation about the SS-bond) and tended to stick to AuNPs surface due to the sulfur atoms in SS- bonds (Fig. 1). Furthermore, when AuNPs were treated with either thiolated or unmodified probes, the SPR peak significantly decreased and the spectrum ranging from 500 to 750 nm. With respect to this issue, the solution color changed from red to pale blue, representing the aggregated AuNPs. However, the rate of aggregation and the corresponding color change were so fast in the AuNPs solution stabilized with unmodified probes. This indicates that, as expected, the thiol-modified probes could protect the AuNPs against aggregation better than unmodified ones.

To further support these observations, the hydrodynamic size and zeta potentials of the DNA- treated AuNPs were evaluated using the dynamic light scattering (DLS) technique. Based on Fig. 2b and c, the mean hydrodynamic diameters of bare AuNPs, AuNPs/self-assembled targets, AuNPs/non-assembled targets, AuNPs/thiolated probes and AuNPs/unmodified probes were approximately 15.2, 68.7, 23.2, 40.4, 28.1 (r.nm) respectively (Fig. 2b,c). These results indicate that the surface of the citrate-stabilized AuNPs was efficiently masked in the case of using self-assembled DNA targets. Accordingly, the average zeta potentials for AuNPs/self-assembled targets and AuNPs/thiolated probes were calculated about −45 mV and −35 mV, respectively, which was significantly higher as compared to that of AuNPs/non-assembled targets (−19.2 mV), and AuNPs/unmodified probes (−26 mV). Based on the DLS analysis, it can be concluded that the self-assembled DNA targets capped AuNPs much better than other DNAs. This fact attributed to longer sizes, flexibility and sulfur rich nature of self-assembled DNA targets (Fig. 2), leading to stronger electrostatic effects and SS-bonds coordinating interactions with the AuNPs surface. As a result, densely coated AuNPs with disulfide-induced assembled dsDNAs can be kept from aggregation at high ionic strengths.

### Experimental evidence

**Figure 3:**
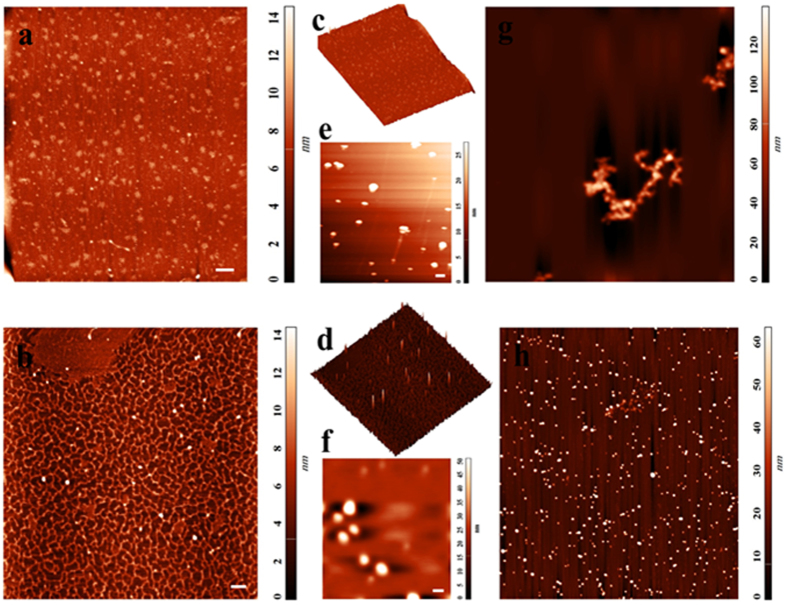
2D and 3D images of DNA by Atomic Force Microscopy (AFM) scanning of mica surface (5 × 5 μm, scale bar: 200 nm) prepared by the solution of thiolated probes (2D: **a**, 3D: **c**) and self assembled DNAs (2D: **b**, 3D: **d**). AFM image of a 2 × 2 μm (scale bar: 100 nm) window of AuNPs incubated with thiolated probes (**e**) and self assembled DNAs (**f**), showing the dimensions difference between particles. Aggregated and dispersed states of AuNPs in blank (**g**) and test sample (**h**) after addition of high salt (10 × 10 μm).

**Figure 4:**
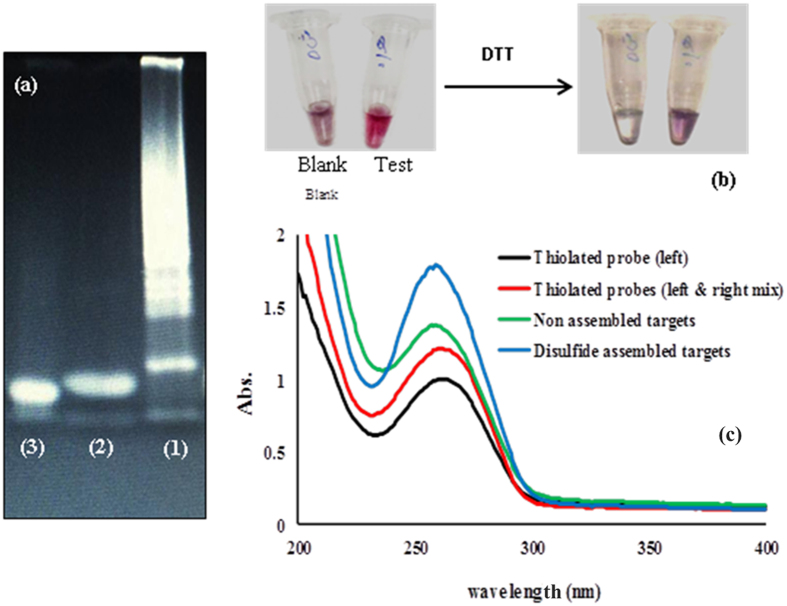
(**a**) Native electrophoresis of assembled DNA products (test sample; lane 1), thiolated-probe mix in the absence of DNA targets (blank sample; lane 2), and DTT-treated assembled DNA products on the polyacrylamide gel (15%). (**b**) Photograph of salt treated AuNPs in the Blank and Test samples after the addition of DTT (1 μM). (**c**) DNA absorption at 260 nm of left/right thiolated -probe mix and assembled targets in comparison with mere left thiol modified probes and non-assembled targets, respectively.

In order to confirm the hypothesis of DNA self assembly in the presence of thiolated probes, and to assess whether stability of modified AuNPs in high salt condition depends upon the self assembeled products scanning probe microscopy with Atomic Force Microscopy (AFM) has been carried out on samples (Fig. 3). AFM spectroscopy is expected to confirm these interpretations by direct imaging of surface topographies. As shown in Fig. 3b,d the AFM results indicate that incubation of DNA target with thiolated probes led to formation of a network of DNA strands with different length. However, in the absence of template (DNA target) thiolated probes did not induce any supermolecular structure and remain barely visible in AFM images (Fig. 3a,c). Then to provide a comparison between two sets of modified AuNPs (Blank & Test sample) “particle analysis” in the AFM software was used to estimate the particles dimensions. As seen in AFM images (Fig. 3e,f), AuNPs are covered with self assembled DNAs have larger dimensions with a mean diameter of 71 nm in comparison to AuNPs in normal conditions (without DNA target, covered with thiolated probes) with a mean diameter of 37 nm. These results are in good agreement with data from our DLS analysis (Fig. 2c), indicating that disulfide rich assembled products were efficiently layered the surface of AuNPs. Also, in view of the more complete shielding of particles in the presence of self assembled DNAs, AuNPs maintain well dispersion in high salt solution without aggregation (Fig. 3h). These results arise from the higher zeta potential of AuNPs being modified with self assembled DNAs, preventing salt induced aggregation due to the repulsion forces. Of note, modification of the AuNPs with thiolated probes, leads to formation of less protective layer of DNA because of a thinner and losser coating, and thus nanoparticle aggregation occurs because of decreased electrostatic repulsion and increased attractive van der Waals forces (Fig. 3g). Afterwards to provide some additional evidence another experiments were conducted to better clarify the basic concept. At first, a polyacrylamide gel of 15% was run to examine whether the continuous disulfide bond formation at 3′ and 5′ ends of DNA targets leads to the formation of different self-assembled structures with different in size (Fig. 4a). As depicted in Fig. 3a (lane 1), a heavy DNA smear, which was an indication of the high amount of DNA fragments with very close molecular weights appeared on the gel for the mixture of thiolated probes and DNA targets. However, when this mixture was treated with Dithiothreitol (DTT) as a reducing agent and subsequently loaded on the gel, only a sharp single band with a length of 30 bp (which was identical to the length of the DNA target) was observed in Fig. 3a (lane 3). In addition, there was no DNA smear in samples containing thiolated probes alone (without DNA target) on the gel (Fig. 4a, lane 2). So, in this respect, strong accordance was found between DNA migration patterns on gel electrophoresis and those obtained by use of AFM imaging (Fig. 3).

In the second experiment, the Blank and Test samples were first treated with a high concentration of salt, and subsequently with 1 μM DTT solution. This led to obvious color changes from pale purple to colorless and red to purple in the Blank and Test solutions, respectively, as shown in the Fig. 3b. Finally, the DNA absorption at 260 nm was measured by UV-Vis spectrophotometry to further provide the evidence on disulfide bond formation between probes as well as between DNA targets recognized by probes (Fig. 4c). Interestingly, the results showed that disulfide-assembled DNA targets had more intense absorption peaks at 260 nm than that of non-assembled targets. Similarly, the mixture of 3′ L and 5′ R probes showed more absorption at 260 nm comparing with 3′ L probe alone (Fig. 4c). These results could indicate that the disulfide bonds absorb light weakly near 260 nm^30^. Thus, the presence of a large number of disulfide-bonds accumulated in solution as results of disulfide induced self-assembly mechanism, resulting in significant increases in the absorption peak at 260 nm (Fig. 4c).

Collectively, the above experiments showed that thiolated probes alone or in the presence of related DNA targets can be easily oxidized to the disulfide in the simple solution (sodium phosphate buffer). The formation of disulfide bonds in the Blank solution produces homodimers/heterodimers species of probes, while different self-assembled fragments of DNA targets are generated in the test solution. In addition, the disulfide induced self-assembling mechanism is reversible and the disulfide bonds can subsequently be reduced back to the thiol by reducing agents.

### Sensitivity and selectivity

**Figure 5:**
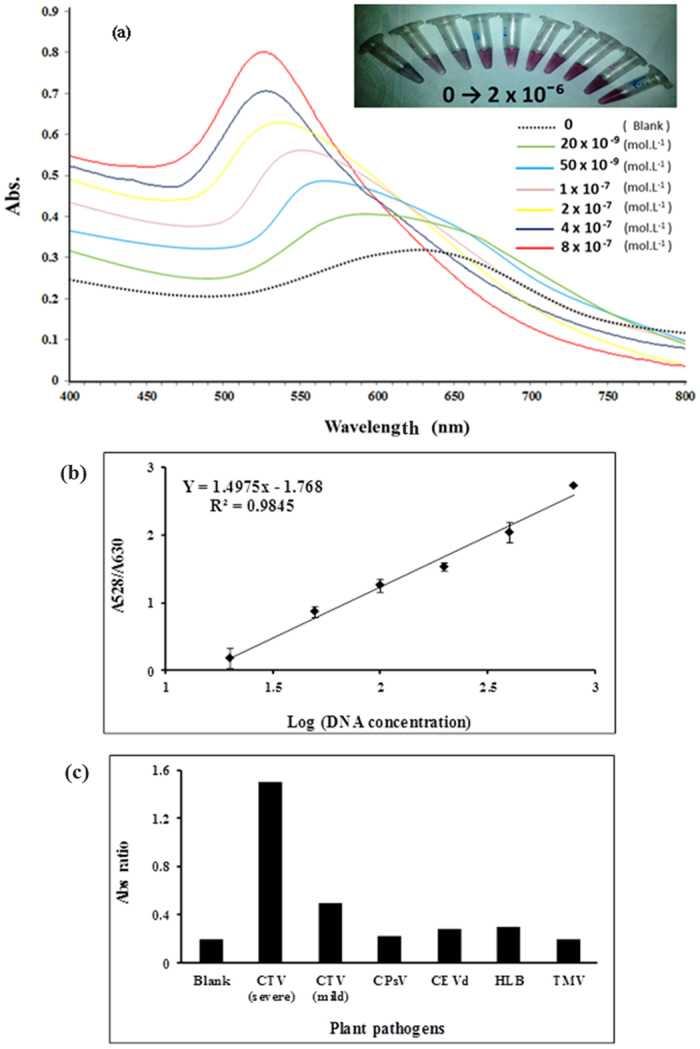
(**a**) Absorption scan of salt-treated AuNPs in response to the different concentration of CTV severe DNA target. *Inset: color change of solutions as seen by naked eye*. (**b**) linear response of the assay calculated based on Abs ratio (A528/A630) plotted versus the logarithmic concentrations of DNA. (**c**) Selectivity of the assay toward CTV severe DNA in comparison with other plant pathogens. The concentration of specific and non-specific targets was 125 ng/ml. Blank: thiolated probes in the absence of targets, CTV (severe): CTV severe strain (specific target), CTV (mild): CTV mild strain, CPsV: Citrus Psorosis Virus, CEVd: Citrus Exocortis Viroid, HLB: citrus greening bacteria, TMV: Tobacco Mosaic Virus.

To determine the sensitivity and diagnostic accuracy of the technique, a series of different concentrations of CTV severe DNA were respectively added and the color changes of the solutions (Fig. 5a, *Inset*) were analyzed. As shown in Fig. 4a, after adjusting the salt levels to 300 mM MgCl_2_ in every sample, the characteristic peak at 528 nm gradually decreased and an obvious red shift (peaked about 630 nm) appeared with the decreasing of the target concentration. Moreover, the SPR band in the Blank sample significantly decreased and widely extended from 500 nm to 750 nm (Fig. 5a). Of note, the absorbance intensity ratio of A 528/A630 was chosen to an acquired calibration curve. As can be seen in Fig. 5a, the absorbance intensity at 528 nm increased proportionally with increasing the concentration from 0 to 8 × 10^−7^ mol.L^−1^. These findings clearly demonstrate that the detection of DNA targets with thiol modified probes via the disulfide self- assembly mechanism causes an increase in the resistance to salt-induced aggregation by concentrating of DNA targets on the AuNPs surface (Fig. 1). Based on this analysis, the linear equation could be fitted as A528/A630 = 1.4975 × − 1.768 (R^2^ = 0.984) with a detection limit of 5.0 × 010^−9^ mol.L^−1^ (Fig. 5b). To evaluate the selectivity of the developed biosensing assay toward the specific target (CTV severe DNA), the values of the absorption ratio (A528/A630) in the presence of some common plant pathogens were measured. The obtained results showed that in the presence of CTV severe targets the absorption ratio value was considerably larger than those of blank or other targets (Fig. 5c). These data indicate that disulfide induced self-assembling based assays display high specificity toward its DNA target. The high selectivity attributed to the specific thiolated probes (left and right) collaboratively triggered the complementary target at both ends. Furthermore, self assembled dsDNAs are effectively formed only when both probes bind to the target.

### Mismatch analysis

**Figure 6:**
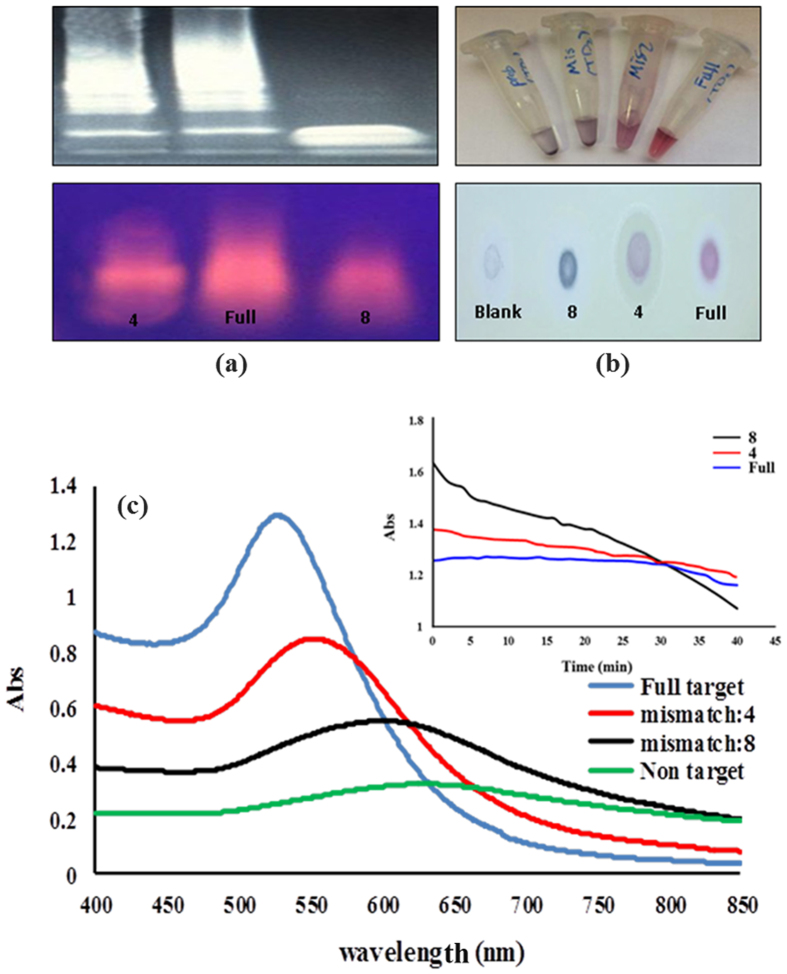
(**a**) UV-Vis spectra of full match target assemblies in comparison with mismatch target assemblies mixed with AuNPs after salt addition. *Inset* aggregation trend of salt-treated AuNPs in the presence full and mismatch targets during the time (40 min), data were acquired at 1 min intervals at 550 nm. (**b**) Mismatch analysis on acrylamide 15% (up) and agarose gel 2.5% (down). (**c**) Representative visual images of full match and mismatch targets mixed with AuNPs after salt addition, in solution (up) and after spotted (10 μl) on the TLC paper (down).

In order to test the ability of the designed assay to track the mismatch targets, the CTV severe DNA sequence was modified in 4 and 8 positions (Table 1). The thiolated probes (3′ L and 5′ R) were then hybridized with a perfect match (CTV severe) or mismatch (4, 8) DNA targets and the final products were visualized using the 15% polyacrylamide or 2.5% agarose gels electrophoresis. Interestingly, as demonstrated in Fig. 6a, a clear difference between sequences could be attained on acrylamide or agarose gels after staining with ethidium bromide. A DNA smear resulted from a product of the disulfide-induced self-assembling process was observed for all sequences, which appears to occur much more easier with full targets than mismatches. In this regard, a heavy DNA smear, which was extended toward higher molecular weights was observed for full DNA targets, while moderate and minimal smears were detected when targets with 4 or 8 mismatches were used respectively (Fig. 6a). Using the proposed novel method, it can be easily distinguish among close DNA sequences even without the addition of AuNPs.

Subsequently, the changes in the optical properties of the AuNPs suspension treated with different type of DNA targets (full and mismatches) were monitored by UV-Vis absorption spectroscopy after salt addition. Figure 6c shows the spectral changes of SPR bands of AuNPs upon the aggregation. The result showed the spectral changes of the treated AuNPs to be sensitive to DNAs type. Hence, under high ionic concentration, the full target/AuNPs suspension showed high stability and remained red with the characteristic surface plasmon band at ∼528 nm (Fig. 6b,c). In contrast, the aggregation of AuNPs treated with 4 and 8 mismatch targets caused a decrease in the intensity and a red shift in the surface plasmon band. In addition, the new LSPR peaks at 560 nm and ∼620 nm appeared in the spectra of AuNPs treated with 4 and 8 mismatch targets respectively (Fig. 6c). These spectral changes co-occurred with a corresponding color change in the solution; varied from red to pale purple to gray-blue as the mismatch positions increased (Fig. 6b). Finally, the perfect match and mismatch DNA targets could be precisely discriminated by following the evolution of AuNPs aggregation versus time after the addition of salts (Fig. 6c, Inset). It was found that the AuNPs protected with assembled full DNA targets exhibited different aggregation behaviors when treated with appropriate concentrations of salt (Fig. 6c, *Inset*). This feature can be potentially used to detect sequences with up to two mismatches (data not shown). Altogether, these results indicate that the approach based on disulfide induced self-assembled targets constitutes a powerful tool for mismatch discrimination analysis. In this regard, such analysis can be sensitively performed by visible absorption monitoring, and easily read out with the naked eye after salt induced aggregation of AuNPs suspension or after gel electrophoresis of self-assembled products.

### Real sample analysis

To survey the analytical performance of the developed colorimetric assay in biological samples, diluted total RNAs (1:5~0.15 μg) extracted from plant and human plasma circulating total RNA was spiked with increasing concentrations of CTV and miRNA18 RNA targets respectively. The hybridization test was conducted by adding specific targets to solutions containing thiolated probes (each of 2 μl), total RNAs (10 μl), phosphate buffer (12 μl, 20 mM) of a final volume of 60 μl. The resulting solutions were then heated to 75 for 5 min and kept at room temperature (4 h) before they were mixed with 90 μl of bare AuNPs. The corresponding data are shown in Table 2, which shows that the present approach could be successfully used for the detection of specific mRNAs from samples as small as 0.1 μg without the need for extra manipulations such as RNA to cDNA conversion and PCR, which are a common step in other methods (Table 2).

## Conclusion

The development of a novel colorimetric approach for the detection of DNA/RNA targets based on unmodified gold nanoparticles was disclosed. In principle, the method is based on the self- assembling of DNA/RNA targets through the continuous formation of disulfide bonds at both ends that was acquired after hybridization with two complementary thiolated probes; each specifically bound at one half of the target. In this approach, the generated products have not only a high affinity for the surface of AuNPs, but also protect them efficiently from salt induced aggregation. Also, this study provides a potential solution to the issues of concern with respect to colorimetric detection of DNAs/RNAs based on typical cross-linking and non-cross-linking aggregation methods. The first key novelty here is that we directly hybridize DNA target with thiolated probes that was lead to SH introduced at both ends of the DNA target. With this simple modification, the conventional labored and time consuming steps, which must be inevitably done during the chemical immobilization of thiolated probes were completely removed. A second key novelty of our proposed system is the ability of small DNA target (30 bp) to assemble at both ends (because of SH groups) and produce long S-rich products. To the best of our knowledge, no similar approach in the field of DNA detection has been previously reported. These products can be tightly deposited on the surface of the AuNPs in solution through SS-bonds coordinating interactions with Au atoms. Thus, in this way the sensitivity of electrostatic based systems of DNA/AuNPs toward the local environment and non-specific response was significantly reduced. More interestingly, this work provides a facile promising method to discriminate full and mismatch sequences by use of gel electrophoresis or through the color signaling of AuNPs suspension. This is reasonable to assume that the strategy of disulfide-induced self assembly of DNA could be potentially applied for further research in areas like gene delivery, enzyme assays, metal ions, proteins, cells or metabolite detection by combining with specific elements, DNAzymes and aptamers.

## Experimental Section

### Materials

Chloroauric acid tetrahydrate (HAuCl_4_.4H_2_O) and trisodium citrate dehydrate were purchased from Sigma and used as received. Other chemicals were of analytical grade and were used without further purification. All oligonucleotides were synthesized and purified with PAGE by BioNEER (Nedayfan co. Iran). The Right and Left probes were chemically thiolated at 5′ site (Right: 5′R) and 3′ site (Left: 3′L) respectively. The sequences and description of all types of oligonucleotides included in our assay were listed in Table 1.

### Instruments

The UV-vis spectra were recorded with a PerkinElmer Lambda 35 spectrophotometer. Transmission electron microscopy (TEM) images were acquired with a JEM 2100F field emission transmission electron microscope. The samples used for the TEM analyses were prepared by placing a drop of a colloidal solution on carbon-coated copper grid and drying it at room temperature. The hydrodynamic diameter of the dispersed and aggregated AuNPs were measured by dynamic light scattering (DLS) (zetasizer, Malvern Instruments Ltd). Native PAGE experiments were carried out in a 0.045 M Triseborate buffer. The electrophoresis was carried out by using 15% acrylamide at 80 V for 120 min at room temperature.

### Preparation of Au Nanoparticles

In brief, a 100 mL solution containing 0.01 g of HAuCl_4_ was boiled, then 2.5 mL of a 1% trisodium citrate solution was quickly added to the HAuCl_4_ solution under vigorous stirring. A few minutes later, the red colloidal suspension was obtained. Then, the mixture solution was kept boiling for 30 min and then cooled to room temperature. The size of the citrate-capped AuNPs determined by TEM image was about 13 nm (data not shown). The particle concentration of the AuNPs (13 nmol L^−1^) was determined according to Beer’s Law using an extinction coefficient of 2.78 × 10^8^ L mol^−1^ cm^−1^ at 520 nm^31^.

### Atomic Force Microscopy (AFM) analysis

AFM imaging was performed on DNAs (probes, self assembled products) and modified AuNPs samples deposited on freshly cleaved mica sheets. For this, 1 × 1 cm mica slides soaked in 5 mM MgCl_2_, 2 min after, the surface was rinsed with ultra-pure distilled water and dried by nitrogen gas. Then, 10 μL of DNAs samples in 1 mM MgCl_2_ and 10 μL of diluted (1:2) modified AuNPs samples (before and after addition of salt) were spotted onto activated mica plates and incubated for 20 min. After washing the samples with deionized water and drying, AFM imaging was done on a Solver PRO AFM system (NT-MDT, Russia), in a semi-contact (tapping) mode, using Si-gold-coated cantilevers (NT-MDT, Zelenograd, Russia) with a resonance frequency of 375 kHz. Images were recorded in height mode, and Nova image processing software (NT-MDT, Zelenograd, Russia) software was used for data processing and particle analysis.

### Detection procedure

Target DNA or RNA samples were mixed with thiolated probes (5′ R, 3′ L) in PBS (20 mM, pH 7.2) and heated to 90 °C for 10 min and then allowed to cool down to room temperature for 4 hr (or O/N) to form disulfide self-assembeled products. Subsequently, 60 μL of the above mixture was added into 90 μL of a solution of AuNPs and after incubation for at least 30 min (or 1 h), some MgCl_2_ (300 mM) was added. The resulting solutions were checked with naked eye and analyzed by UV-Vis. The calibration curve was plotted by measuring the ratio of UV/Vis absorbances between 528 and 630 nm (A528/A630) versus the different concentrations of targets.

### Preparation of the biological sample

Human plasma was acquired from Iranian Blood Transfusion Organization. 2 ml aliquots from three healthy donors were used for human plasma circulated total RNA isolation by using the Stratagene total RNA kit according to the manufacturer’s instructions with minor modifications. Finally, plasma circulating total RNA was eluted in 50 μl of H_2_O and different concentrations of target RNA (miRNA18) were spiked for analysis. Similarly, the total RNA was extracted from healthy citrus leaves using TRIZOL method and *Tristeza* virus RNA was manually added.

## Additional Information

**How to cite this article:** Shokri, E. *et al*. Disulfide-induced self-assembled targets: A novel strategy for the label free colorimetric detection of DNAs/RNAs via unmodified gold nanoparticles. *Sci. Rep.* **7**, 45837; doi: 10.1038/srep45837 (2017).

**Publisher's note:** Springer Nature remains neutral with regard to jurisdictional claims in published maps and institutional affiliations.

## Figures and Tables

**Table 1:** List of probes and oligo strands used in this study.

Label	Type	bp	Seq (5′ … 3′)	Description	Use
5′ R (CTV)	ssDNA	15	SH-(CH2) 6-ACTATCAACCTGTCG	5′ thiolated probe, bind to target on the right half	Colorimetric
3′ L (CTV)	ssDNA	15	CTAAGTCATCCGCTT (CH2) 6–SH	3′ thiolated probe, bind to target on the left half	Colorimetric
5′ UR (CTV)	ssDNA	15	ACTATCAACCTGTCG	5′ unmodified probe, bind to target on the right half	Colorimetric
3′ UL (CTV)	ssDNA	15	CTAAGTCATCCGCTT	3′ unmodified probe, bind to target on the left half	Colorimetric
5′ R (miRNA18)	ssDNA	11	SH-(CH2) 6-CTATCTGCACT	5′ thiolated probe for miRNA18	Recovery
3′ L (miRNA18)	ssDNA	11	AGATGCACCTT (CH2) 6–SH	3′ thiolated probe for miRNA18	Recovery
Full target	ssDNA	30	AAGCGGATGACTTAGCGACAGGTTGATAGT	Citrus Teristeza severe strain	Colorimetric
Mismatch 4	ssDNA	30	AAGCGGATGACTTAGCGACAGGTTGATAGT	Modified full target strand with 4 mismatch	Mismatch
Mismatch 8	ssDNA	30	AAATGGACGACTTGATGACTGGTTGATCGT	Modified full target strand with 8 mismatch	Mismatch
miRNA18a	RNA	22	AAGGUGCAUCUAGUGCAGAUAG	Breast specific miRNA18	Recovery
Mild	ssDNA	30	AAATGGACGACTTGATGACTGGTTGATCGT	Citrus Teristeza mild strain	Selectivity
CpsV	ssDNA	30	GAAAGGAGACACTAAATCACTCAACTTGTT	Citrus Psorosis Virus	Selectivity
CEVd	ssDNA	30	TCTTTCTTGAGGTTCCTGTGGTGCTCACCT	Citrus Exocortis Viroid	Selectivity
HLB	ssDNA	30	AATTACGGCGAAGAGGAAGACTCCGATATC	Citrus greening bacterium	Selectivity
TMV	ssDNA	30	GGAAGACCATACCGACCGCTACAGTTAGAT	Tobacco Mosaic Virus	Selectivity

**Table 2:** The recovery of CTV severe RNA and miRNA18 subjected to the different concentrations in real samples.

Targets	Media	Added (mol/L^−1^)	Detect Mean SD (mol/L^−1^)	Recovery %
CTV-RNA	Citrus Total RNA	4.3 × 10^−7^	3.78 × 10^−7^ ± 4.3 × 10^−8^	88
CTV-RNA	Citrus Total RNA	13 × 10^−7^	11.98 × 10^−7^ ± 7.3 × 10^−8^	92.2
miRNA18	Human Plasma Circulating Total RNA	4.3 × 10^−7^	3.51 × 10^−7^ ± 6.5 × 10^−8^	81.6
miRNA18	Human Plasma Circulating Total RNA	13 × 10^−7^	11.58 × 10^−7^ ± 5.1 × 10^−8^	89.1

## References

[b1] Hunter R. J. Foundations of Colloid Science Oxford University Press Incorporated (2001).

[b2] Elghanian R., Storhoff J. J., Mucic R. C., Letsinger R. L. & Mirkin C. A. Selective Colorimetric Detection of Polynucleotides Based on the Distance-Dependent Optical Properties of Gold Nanoparticles. Science 277, 1078–1081 (1997). 9262471 10.1126/science.277.5329.1078

[b3] Kaminker R., Lahav M., Motiei L., Vartanian M., Popovitz-Biro R., Iron M. A. & van der Boom M. E. Molecular Structure–Function Relations of the Optical Properties and Dimensions of Gold Nanoparticle Assemblies. Angew. Chem. 122, 1240–1243 (2010). 10.1002/anie.200906636 20108293

[b4] Devi R. V., Doble M. & Verma R. S. Nanomaterials for early detection of cancer biomarker with special emphasis on gold nanoparticles in immunassays/sensors. Biosens. Bioelectron. 68, 688–698 (2015). 25660660 10.1016/j.bios.2015.01.066

[b5] Noguez C. Surface plasmons on metal nanoparticles: the influence of shape and physical environment. J. Phys. Chem. C 111, 3806–3819 (2007).

[b6] Cao X., Ye Y. & Liu S. Gold nanoparticle-based signal amplification for biosensing. Anal. biochem. 417, 1–16 (2011). 21703222 10.1016/j.ab.2011.05.027

[b7] Hutter E. & Maysinger D. Gold-nanoparticle-based biosensors for detection of enzyme activity. Trend. Pharmacol. Sci. 34, 497–507 (2013). 10.1016/j.tips.2013.07.002 23911158

[b8] Zhao W., Brook M. A. & Li Y. F. Design of Gold Nanoparticle-Based Colorimetric Biosensing Assays. ChemBioChem. 9, 2363–2371 (2008). 18821551 10.1002/cbic.200800282

[b9] Ghosh S. K. & Pal T. Interparticle Coupling Effect on the Surface Plasmon Resonance of Gold Nanoparticles: From Theory to Applications. Chem. Rev. 107, 4797–4862 (2007). 17999554 10.1021/cr0680282

[b10] Mirkin C. A., Letsinger R. L., Mucic R. C. & Storhoff J. J. A DNA-based method for rationally assembling nanoparticles into macroscopic materials. Nature 382, 607–609 (1996). 8757129 10.1038/382607a0

[b11] Cao Y. C., Jin R. & Mirkin C. A. Nanoparticles with Raman spectroscopic fingerprints for DNA and RNA detection. Science 297, 1536–1540 (2002). 12202825 10.1126/science.297.5586.1536

[b12] Saha K., Agasti S. S., Kim C., Li X. & Rotello V. M. Gold nanoparticles in chemical and biological sensing. Chem. Rev. 112, 2739–2779 (2012). 22295941 10.1021/cr2001178 PMC4102386

[b13] Lim I. I. S., Chandrachud U., Wang L., Gal S. & Zhong C. J. Assembly− Disassembly of DNAs and Gold Nanoparticles: A Strategy of Intervention Based on Oligonucleotides and Restriction Enzymes. Anal. Chem. 80, 6038–6044 (2008) 18613651 10.1021/ac800813a

[b14] Xu X., Han M. S. & Mirkin C. A. A Gold‐Nanoparticle‐Based Real‐Time Colorimetric Screening Method for Endonuclease Activity and Inhibition. Angew. Chem. 119, 3538–3540 (2007). 10.1002/anie.200605249 17385814

[b15] Xu X., Daniel W. L., Wei W. & Mirkin C. A. Colorimetric Cu^2+^ Detection Using DNA‐Modified Gold‐Nanoparticle Aggregates as Probes and Click Chemistry. Small 6, 623–626 (2010). 20108231 10.1002/smll.200901691 PMC3517019

[b16] Lee J. S., Ulmann P. A., Han M. S. & Mirkin C. A. A DNA-gold nanoparticle-based colorimetric competition assay for the detection of cysteine. Nano Lett. 8, 529–533 (2008). 18205426 10.1021/nl0727563

[b17] Trantakis I. A., Bolisetty S., Mezzenga R. & Sturla S. J. Reversible aggregation of DNA-decorated gold nanoparticles controlled by molecular recognition. Langmuir 29, 10824–10830 (2013). 23883185 10.1021/la401211u

[b18] Lam M. K., Gadzikwa T., Nguyen T., Kausar A., Alladin-Mustan B. S., Sikder M. D. & Gibbs-Davis J. M. Tuning Toehold Length and Temperature to Achieve Rapid, Colorimetric Detection of DNA from the Disassembly of DNA–Gold Nanoparticle Aggregates. Langmuir 32, 1585–1590 (2016). 26707736 10.1021/acs.langmuir.5b03777

[b19] Li H. X. & Rothberg L. Colorimetric detection of DNA sequences based on electrostatic interactions with unmodified gold nanoparticles. Proc. Natl. Acad. Sci. USA 101, 14036 (2004). 15381774 10.1073/pnas.0406115101 PMC521116

[b20] Li H. & Rothberg L. J. Label-free colorimetric detection of specific sequences in genomic DNA amplified by the polymerase chain reaction. J. Am. Chem. Soc. 126, 10958–10961 (2004). 15339181 10.1021/ja048749n

[b21] Li H. & Rothberg L. Detection of specific sequences in RNA using differential adsorption of single-stranded oligonucleotides on gold nanoparticles. Anal. Chem. 77, 6229–6233 (2005). 16194083 10.1021/ac050921y

[b22] Valentini P. & Pompa P. P. Gold nanoparticles for naked-eye DNA detection: smart designs for sensitive assays. RSC Adv. 3, 19181–19190 (2013).

[b23] Lee H., Joo S. W., Lee S. Y., Lee C. H., Yoon K. A. & Lee K. Colorimetric genotyping of single nucleotide polymorphism based on selective aggregation of unmodified gold nanoparticles. Biosens. Bioelectron. 26, 730–735 (2010). 20674325 10.1016/j.bios.2010.06.050

[b24] Xia F., Zuo X., Yang R., Xiao Y., Kang D., Vallée-Bélisle A., Gong X., Yuen J. D., Hsu B. B., Heeger A. J. & Plaxco K. W. Colorimetric detection of DNA, small molecules, proteins, and ions using unmodified gold nanoparticles and conjugated polyelectrolytes. Proceed. National Academy Sci. 107, 10837–10841 (2010). 10.1073/pnas.1005632107 PMC2890709 20534499

[b25] Storhoff J. J., Elghanian R., Mucic R. C., Mirkin C. A. & Letsinger R. L. One-pot colorimetric differentiation of polynucleotides with single base imperfections using gold nanoparticle probes. J. Am. Chem. Soc. 120, 1959–1964 (1998).

[b26] Zhou Z., Wei W., Zhang Y. & Liu S. DNA-responsive disassembly of AuNP aggregates: influence of nonbase-paired regions and colorimetric DNA detection by exonuclease III aided amplification. J. Mater. Chem. B 1, 2851–2858 (2013). 10.1039/c3tb20206b 32260871

[b27] Song Y., Wei W. & Qu X. Colorimetric Biosensing Using Smart Materials. Adv. Mater. 23, 4215–4236 (2011). 21800383 10.1002/adma.201101853

[b28] Niblett C. L. Genc H., Cevik B., Halbert S., Brown L., Nolasco G., Bonacalza B., Manjunath K. L., Febres V. J., Pappu H. R. & Lee R. F. Progress on strain differentiation of Citrus tristeza virus and its application to the epidemiology of citrus tristeza disease. Virus Res. 71, 97–106 (2000). 11137165 10.1016/s0168-1702(00)00191-x

[b29] Hilf M. E., Mavrodieva V. A. & Garnsey S. M. Genetic Marker Analysis of a Global Collection of Isolates of Citrus tristeza virus: Characterization and Distribution of CTV Genotypes and Association with Symptoms. Phytopathology 95, 909–917 (2005). 18944413 10.1094/PHYTO-95-0909

[b30] Schmid F. X. Biological Macromolecules: UV‐visible Spectrophotometry. Encycl. Life Sci. 1–4 (2001).

[b31] Jin R., Wu G., Li Z., Mirkin C. A. & Schatz G. C. What Controls the Melting Properties of DNA-Linked Gold Nanoparticle Assemblies? J. Am. Chem. Soc. 125, 1643–1654 (2003). 12568626 10.1021/ja021096v

